# Supplementary material for: Whole-genome Duplications and the Long-term Evolution of Gene Regulatory Networks in Angiosperms
Source: Mol Biol Evol. 2023 Jul 5;40(7):msad141. doi: 10.1093/molbev/msad141 (PMC10321489; doi:10.1093/molbev/msad141)
Supplement: msad141_Supplementary_Data [file msad141_supplementary_data.zip › Supplementary_Figures_MBE-23-0212.pdf]

Supplementary Figures

Whole-genome duplications and the long-term evolution of gene regulatory networks in angiosperms

Fabricio Almeida-Silva[1,2] and Yves Van de Peer[1,2,3,4*]

[1] Department of Plant Biotechnology and Bioinformatics, Ghent University, 9052 Ghent, Belgium
[2] VIB Center for Plant Systems Biology, VIB, 9052 Ghent, Belgium
[3] Centre for Microbial Ecology and Genomics, Department of Biochemistry, Genetics and Microbiology, University of Pretoria, Pretoria 0028, South Africa.
[4] College of Horticulture, Academy for Advanced Interdisciplinary Studies, Nanjing Agricultural University, Nanjing, China.

[*] To whom correspondence should be addressed (yves.vandepeer@psb.vib-ugent.be).

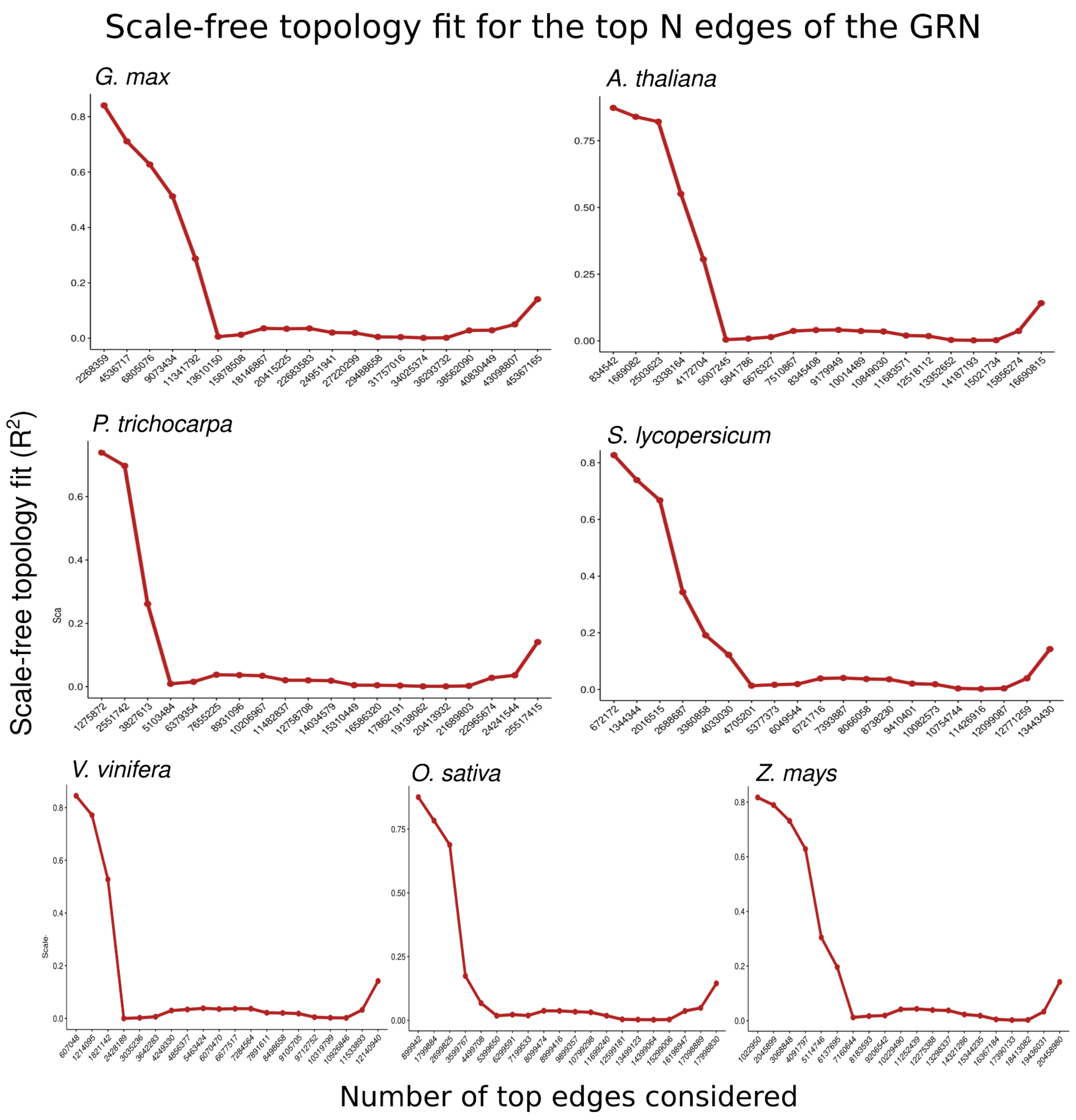

Fig. S1. Scale-free topology fit for each subset of the fully connected gene regulatory networks. For each species, subnetworks were created by ranking and extracting the top *N* edges (x axis). The original fully connected graph for each species does not satisfy the scale-free topology fit, but subsets of the top-ranked edges do. The highest value of *N* for which the network satisfied the scale-free topology fit (R^2 = 0.75) was selected as optimal

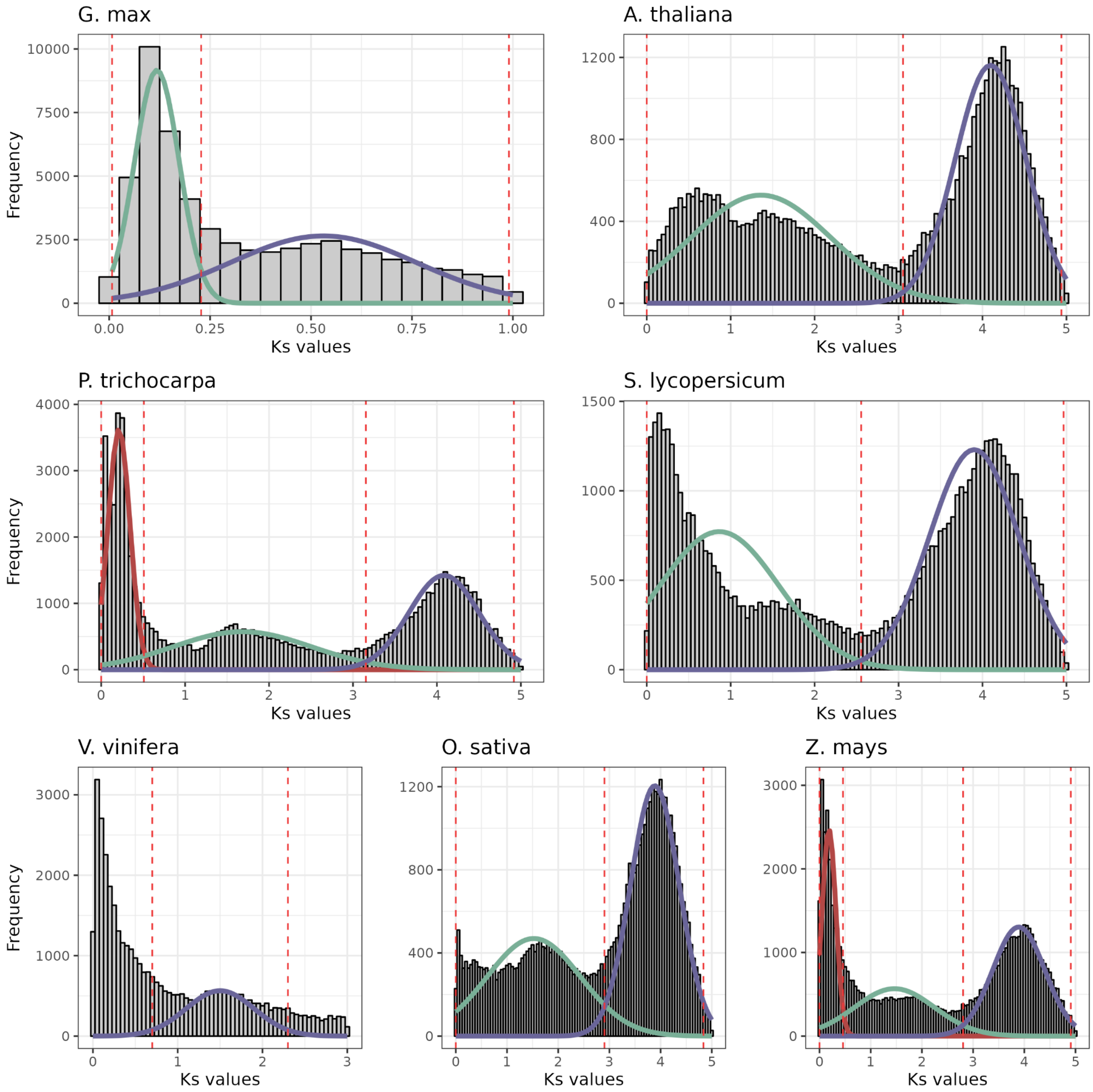

Fig. S2. Peaks in K_s distributions for each species and age boundaries. Density lines represent Ks peaks identified with Gaussian mixture models. Dashed red lines represent peak-based age boundaries, which were used to split duplicated gene pairs in age groups. Only WGD- and SSD-derived gene pairs from the same age group were compared, as age can be a confounder when comparing motif frequencies.

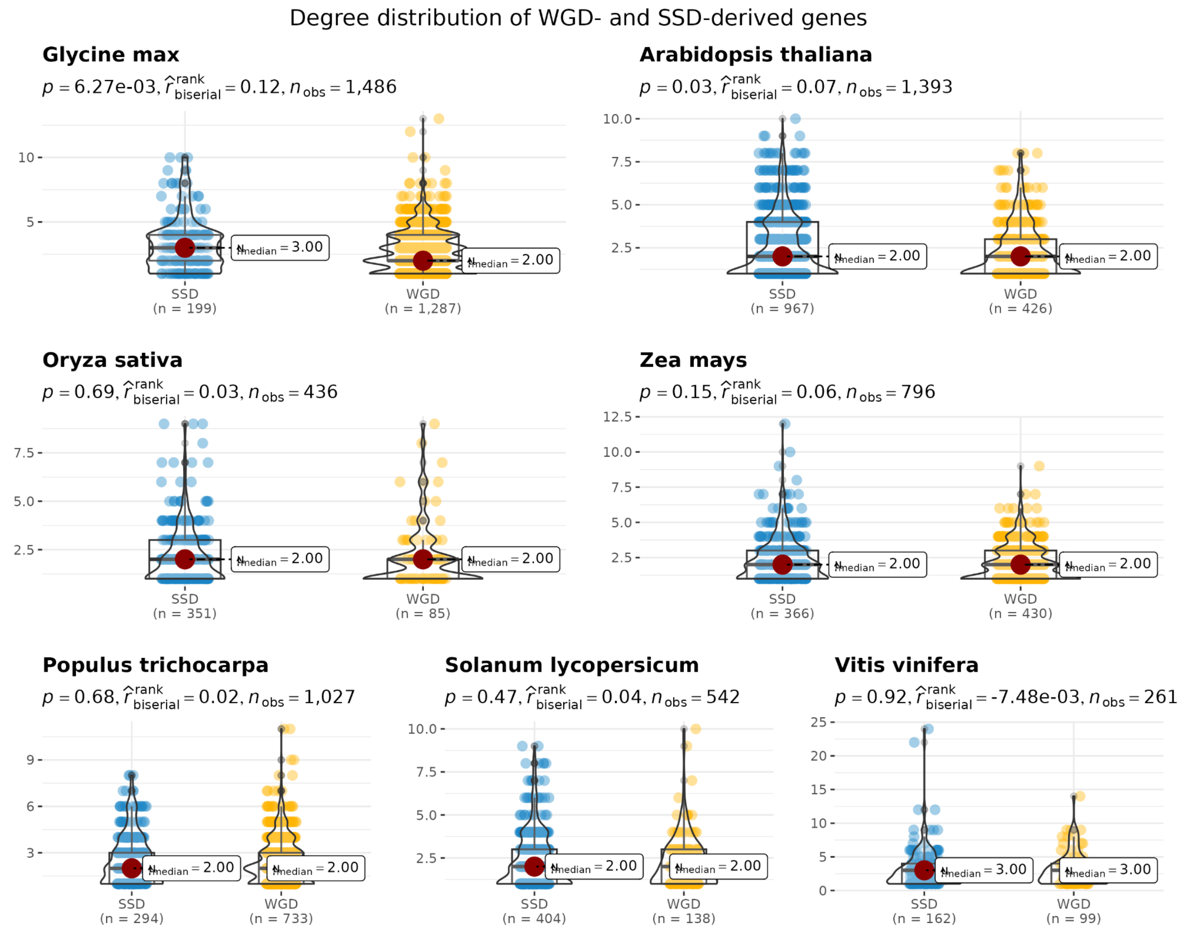

Fig. S3. Comparison of the degree distributions for WGD- and SSD-derived genes in PPI networks. The Mann-Whitney U test revealed no differences in degree distributions. Although some comparisons showed significant differences ($P < 0.05$), the effect size is negligible (rank-biserial correlation < 0.15), suggesting that the low *P*-values are likely an artifact resulting from large sample sizes.

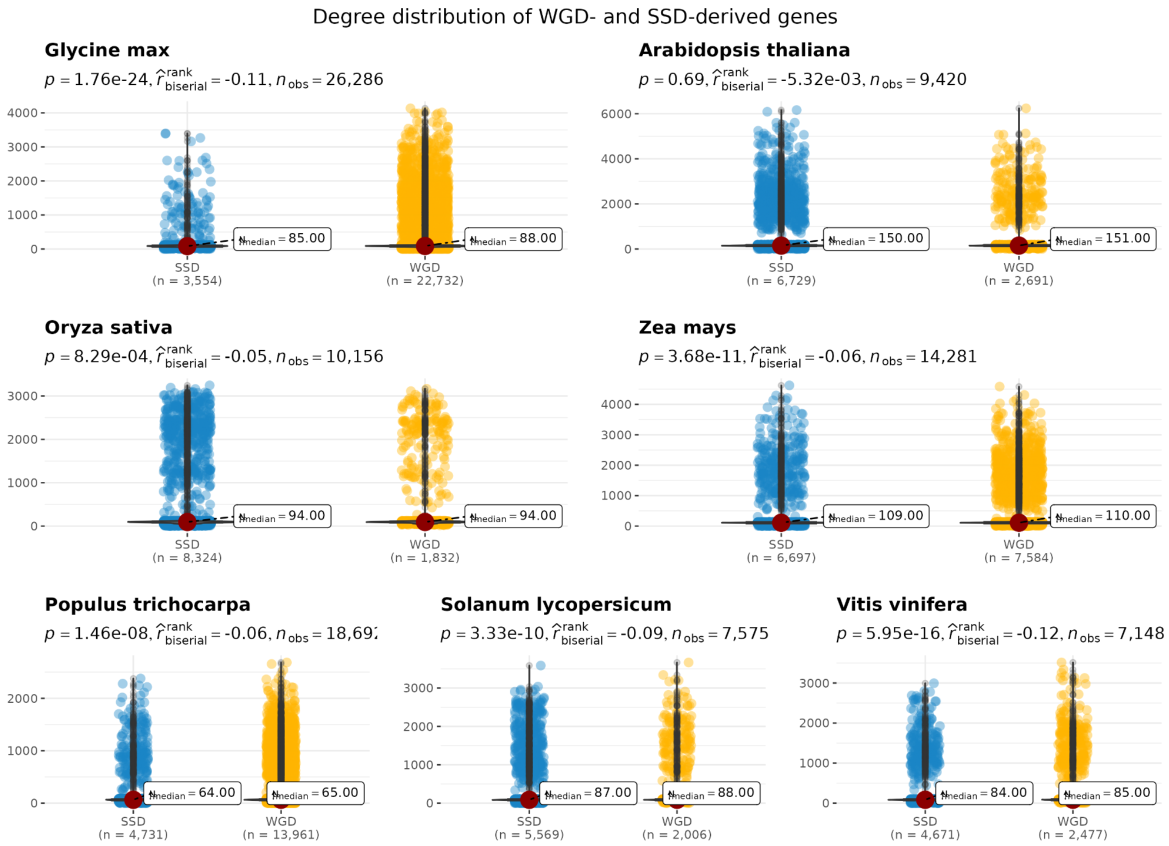

Fig. S4. Comparison of the degree distributions for WGD- and SSD-derived genes in GRNs. There were no differences in degree distributions (Mann-Whitney U test). Although some comparisons showed significant differences ($P < 0.05$), the effect size is negligible (rank-biserial correlation < 0.15), suggesting that the low *P*-values are likely an artifact resulting from large sample sizes.

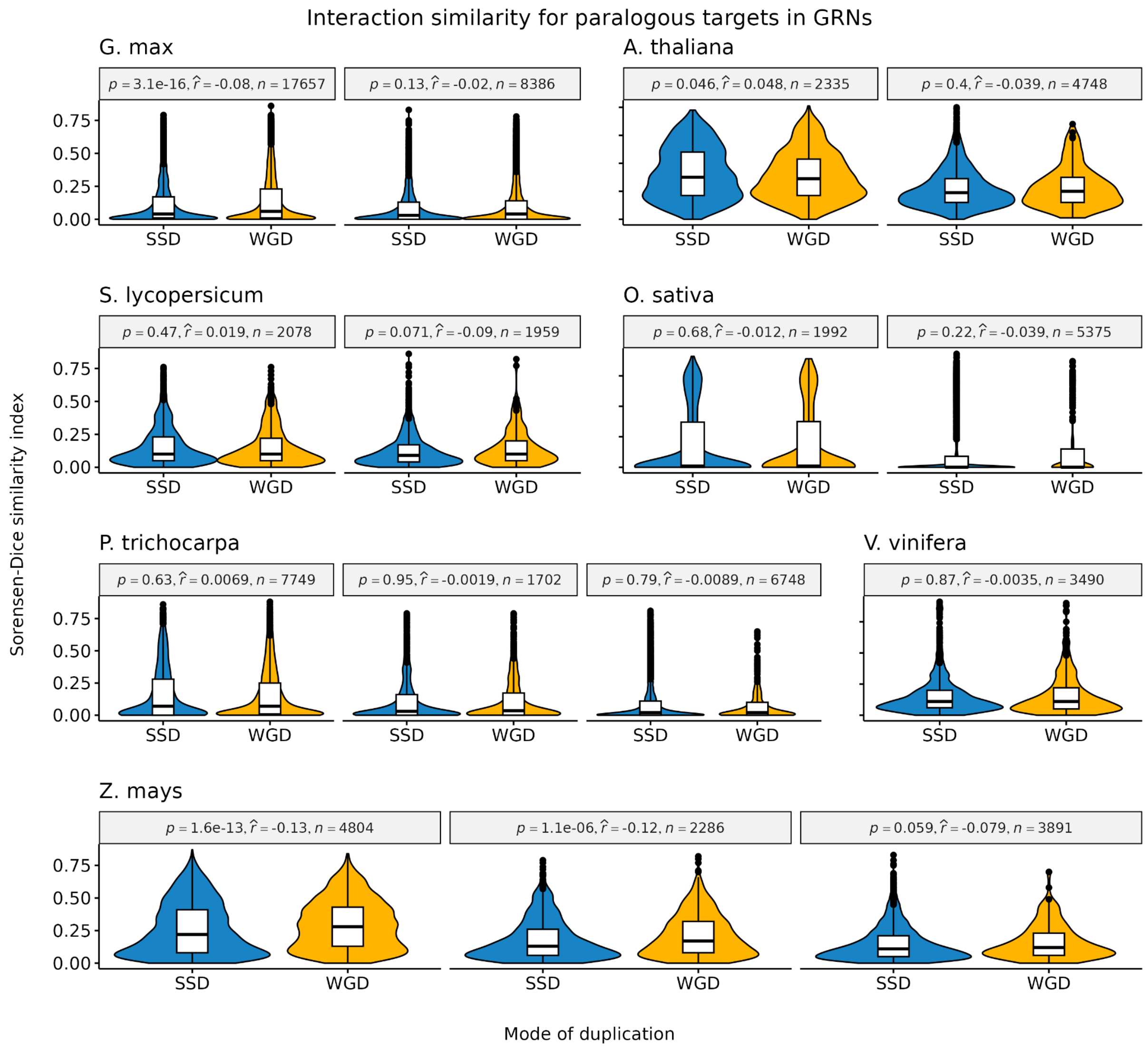

Fig. S5. Interaction similarity between paralogous target genes in GRNs. Sorensen-Dice similarity indices were used to indicate interaction similarity. No differences were observed between WGD- and SSD-derived gene pairs (Mann-Whitney U test; $P < 0.05$). Although some comparisons had significant *P*-values, it is likely an artifact resulting from large sample sizes, as effect sizes are negligible.

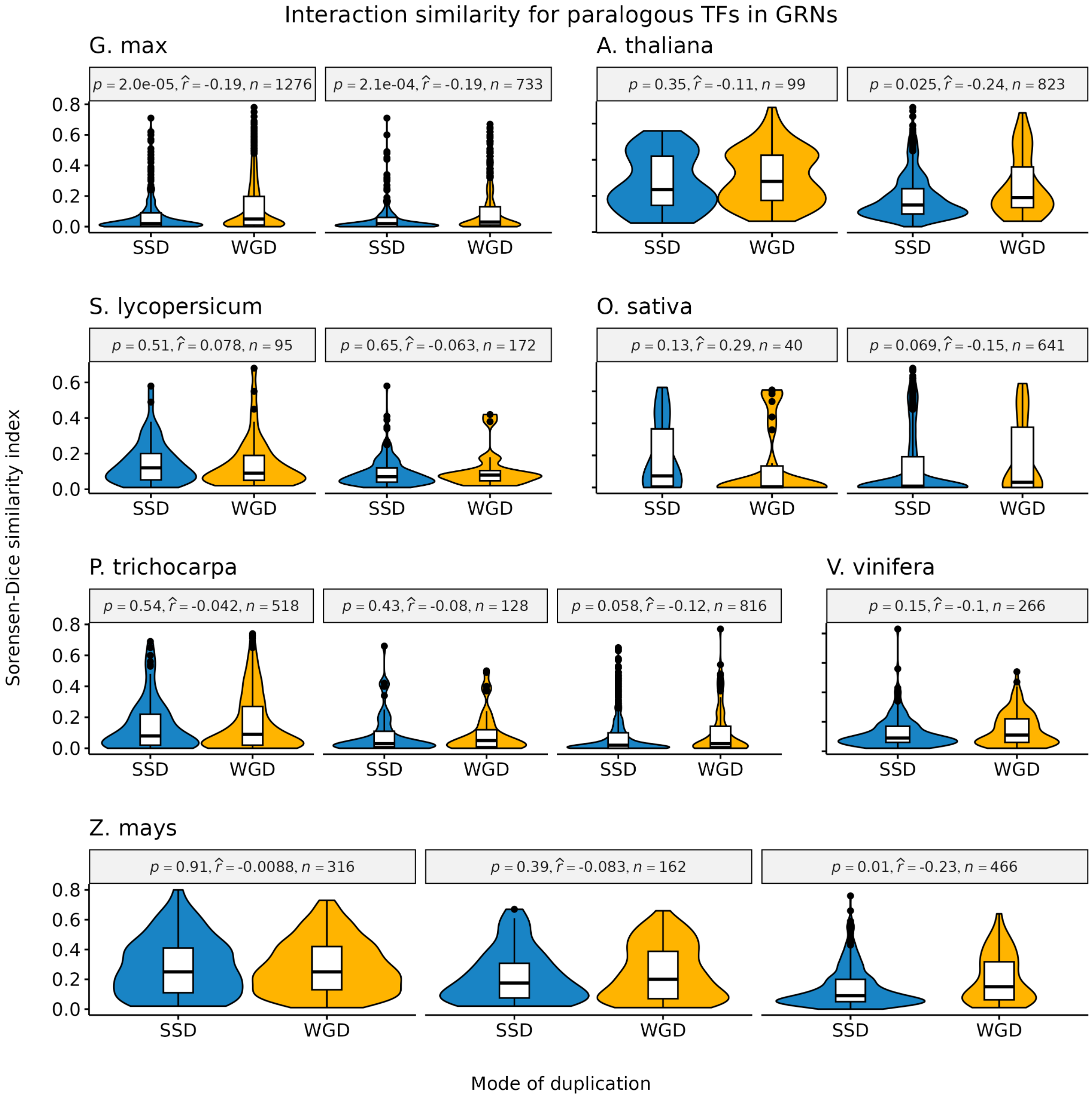

Fig. S6. Interaction similarity between paralogous TFs in GRNs. Sorensen-Dice similarity indices were used to indicate interaction similarity. Overall, no differences were observed between WGD- and SSD-derived gene pairs (Mann-Whitney U test; $P < 0.05$). Although some comparisons had significant *P*-values, it is likely an artifact resulting from large sample sizes, as effect sizes are negligible.
